# Intestinal parasites in rodents from five different Himas in Lebanon

**DOI:** 10.2478/helm-2025-0011

**Published:** 2025-09-30

**Authors:** M. Abi Said, T. Maroun, H. Shaib

**Affiliations:** 1 L2GE, Department of Earth and Life Sciences, Faculty of Sciences II, Lebanese University, Fanar, Lebanon; 2 Department of Agriculture, Faculty of Agricultural and Food Sciences, American University of Beirut, Lebanon

**Keywords:** Intestinal parasites, rodents, zoonosis, Himas, Lebanon

## Abstract

Rodent and their parasites serve as a reliable indicator of ecosystem health, which is critical in determining the structure of ecological communities. Therefore, gaining a thorough comprehension of the diversity of intestinal parasites and the factors infl uencing their interaction is of primary importance. This study assesses the diversity of gastrointestinal parasites in wild rodents in Lebanon. Two thousand fi ve hundred trapping nights were carried out in fi ve Himas in Lebanon during the spring and fall of 2022. A total of 205 rodents, including *Apodemus mystacinus, Apodemus flavicollis, Apodemus hermonensis, Microtus guentheri, Rattus rattus*, and *Mus musculus*, were trapped and examined for intestinal parasites. Nine intestinal parasites were isolated, including one cestode species, *Hymenolepis diminuta* (11.7 %), and eight nematodes: *Ascaris lumbricoides* (31.7 %), *Trichuris muris* (7.8 %), *Aspiculuris tetraptera* (7.3 %), *Heligmosomoides polygyrus* (6.8 %), *Syphacia muris* (3.9 %), *Syphacia obvelata* (3.4 %), *Capillaria spp*. (2.4 %), and *Physaloptera spp* (0.5 %). The species and gender did not infl uence the number of rodents infected with these parasites. However, the season impacted the number of rodents infected with *A. lumbricoides, T. muris*, and *S. muris*. Infection of rodents with *H. diminuta* and *A. lumbricoides* was infl uenced by the site of trapping. Among the collected parasites, *A. lumbricoides, H. diminuta*, and *Syphacia obvelata* are considered zoonotic. This study showed that preserving parasite-host dynamics and biological cycles depends heavily on environmental stability. This project will further advance the understanding of rodent parasites and support future studies on endoparasites in Lebanon and the region.

## Introduction

Biodiversity is the variability among living organisms from all sources and the ecological complexes of which they are part (Verma, 2016). The Mediterranean region, including Lebanon, is considered a biodiversity hotspot (Myers *et al*., 2000) and is home to numerous plants, amphibians, reptiles, birds, and mammal species. Anthropogenic activity negatively impacts biodiversity through overpopulation, overexploitation, habitat destruction, pollution, introduction of invasive species, and climate change (Prakash & Verma, 2022). While these activities may contribute to increased population densities in some small mammals such as rodents (Korpimäki & Krebs, 1996), they can simultaneously threaten endangered species by degrading their habitats and disrupting ecological balance.

Rodents comprise the largest group of mammals in terms of total species, accounting for more than 40 % of all mammalian species (Mammal Diversity Database, 2018). Many species of rodents can contribute to ecosystem function and are important indicators of environmental changes. Rodents are well-known hosts and reservoirs for various intestinal parasites that can profoundly influence ecosystems. These parasites are referred to as ecosystem engineers because they have the potential to alter the physiological conditions of rodent populations, such as their health and behavior, thereby directly or indirectly modifying resource availability for other species within the ecosystem. For example, parasitic infections may impair a rodent’s foraging ability, affecting its survival and predator-prey dynamics within its habitat (Poulin, 1999). While zoonoses are important in this context, our primary focus is on how these interactions shape ecological relationships (Rabiee *et al*., 2018).

The helminths of rodents have been studied in various parts of the world (Moradpour *et al*., 2018; Adu-Gyasi *et al*., 2018; Ahmad *et al*., 2014; Kataranovski *et al*., 2011). The component structure of helminth communities in wild rodents in any geographical region is influenced by both extrinsic (year, season, site) and intrinsic (host sex, age, reproductive status) factors, but this influence can vary significantly across different geographical regions (Woolhouse, 1998; Zuk & McKean, 1996). For instance, in North America, studies have shown that seasonal variations affect the prevalence and diversity of helminths in rodent populations, with higher infection rates observed during warmer months (Preisser, 2019). In contrast, European research has highlighted the role of host age and reproductive status as critical intrinsic factors influencing helminth community composition, with older rodents exhibiting higher susceptibility to infections (Behnke *et al*., 1999). Furthermore, a study in South America demonstrated that environmental conditions such as habitat type and climate also play a crucial role in shaping helminth communities among local rodent species (Riquelme *et al*., 2021). In Addition, the health of the host species (Johnson & Hoverman, 2012), behavior (Herbison *et al*., 2018), sexual selection (Auld *et al*., 2016), and population control (Dobson, 1988) can all be impacted by the diversity of parasite species and the degree of infection.

This study investigates the prevalence of helminthic parasites among different rodent species in various Lebanese Himas areas designated for conserving natural resources, including fields, wildlife, and forests. This work has two primary objectives: 1) to assess the prevalence of gastrointestinal helminth species in wild rodents from five different Himas in Lebanon, and 2) to investigate the effect of host-related (gender, species) and extrinsic factors (season, site) on helminth prevalence in these rodents.

## Materials and Methods

### Study area

**Fig. 1.**
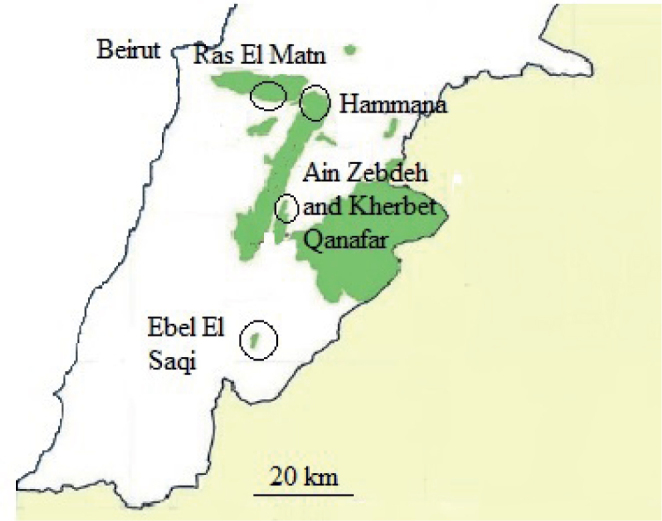
The five Lebanese Hima sites - Kherbet Qanafar, Ras El Matn, Ain Zebdeh, Ebel El Saqi, and Hammana

The five Lebanese Hima sites - Kherbet Qanafar, Ras el Matn, Ain Zebdeh, Ebel el Saqi, and Hammana (Fig. 1) - represent Lebanon’s significant ecological and cultural heritage areas. These sites are part of a traditional resource management system known as “Hima,” which emphasizes sustainable use of natural resources while preserving biodiversity. Each site showcases unique landscapes and ecosystems, contributing to the environmental richness of Lebanon.

#### a. Ras El Matn

Ras El Matn Hima is a mixed deciduous and evergreen (pine oaks) forest, characterized by the co-occurrence of *Calliprinos* pine and oak trees, situated on a steep, thick limestone shelf slope, overlooking the Beirut River Valley, also known as the “Lamartine Valley”. The forest is divided into two main zones: the upper virgin and reforested woodland and the lower degraded forest and scrubland. The Hima is situated at an elevation between 400 and 700 meters above sea level. Ras El Matn Hima is considered a biodiversity hotspot, with many mammal species, including insectivores, bats, carnivores, and rodents. The presence of abundant resources, including water and forage, combined with various microhabitats, contributes to the diversity of the mammal fauna (MESD, 2025; SPNL, 2018).

#### b. Hammana

Hammana Hima is a stunning and unique ecosystem. It is situated on a sandstone ridge, and it overlooks the village, located on the western borders of Jabal Kneisseh, one of the summits of Mount Lebanon, and just south of Jabal Sannine. It is situated at an elevation ranging between 1300 and 1550 meters above sea level. The location is characterized by a coniferous cedar-pine forest, which provides shelter for numerous bird species and other animals. The mountain chain is home to an array of plant species and endemism. Hammana Hima features various transects, such as slopes, trails, woodlands, and quadrats, including woodland, forest clearings, and pastures. The site is rich in avifauna and is significant for floristic, entomological, and herpetological biodiversity (SPNL, 2019).

#### c. Kherbet Qanafar-Ain Zebdeh

Kherbet Qanafar-Ain Zebdeh Hima is a unique and diverse ecosystem located on the eastern slopes of Mount Lebanon and Jabal Barouk. The site is approximately 1,200 meters above sea level, making it a Supramediterranean site, and its geological composition is characterized by thick shelf limestone. This combination of elevation and geological composition creates a range of soil types and conditions that support a variety of plant and animal life. The Hima is a mixed broadleaf forest that covers approximately 600 hectares and is part of one of Lebanon’s largest remaining natural forest areas. The forest is dominated by oak and pine trees and includes several distinct habitats, each with its unique features and characteristics. The Hima’s mixed broadleaf forest also includes scrubland areas characterized by sparse vegetation and rocky outcroppings. The site’s riparian zone includes the Ain Zebdeh and Kherbet Qanafar rivers, which support a rich and diverse array of aquatic and semi-aquatic species. The agricultural land within the Hima includes orchards and fields of wheat and barley that provide important habitat and food sources for the surrounding biodiversity (Sakr, 2023; SPNL, 2014).

#### d. Ebel El Saqi

Ebel El Saqi Hima is a complex and diverse ecosystem located on the western slopes of Mount Hermon. The site is situated at an elevation of approximately 700 meters above sea level, making it an Eumediterranean site. The geological composition of the site is characterized by a combination of volcanic basalts, chalks, limestone substrates, and sandstones overlain by thick shelf limestones, creating a range of soil types and conditions that support a variety of plant and animal life. The 38-hectare pine woodland is a dominant feature of the ecosystem, providing critical habitat for a wide range of plant and animal species. The riverine ecotone is a crucial ecosystem feature, providing a vital habitat for aquatic and semi-aquatic species. The riparian zone along the Hasbani River and its tributaries supports a rich and diverse array of plant and animal life, including species such as the Syrian spiny-tailed lizard and the common kingfisher. The agricultural land within the Hima includes olive groves and grain fields that provide important habitats and food sources for various species. These habitats are particularly important for species adapted to human-dominated landscapes, such as many bird species that rely on agricultural fields for foraging and nesting (SPNL, 2025).

### Sampling

Sampling was conducted in two seasons, Spring (May and June 2022) and Autumn (September 2022). Twenty-seven trapping stations were deployed for five consecutive nights and covered the different landscapes/habitats present in the Himas. The stations were distributed as follows among the five Himas: 6 stations in Ras El Maten, 6 stations in Hammana, 4 stations in Kherbet Qanafar, 6 stations in Ain Zebdeh, and 5 in Ebel El Saqi. In each station, 10 Sherman® live rodent traps spaced 2 – 4m apart were set at dawn and were checked the next day at dusk in locations close to identified burrows or suitable habitats. The traps were baited with peanut butter, cucumber, sunflower seeds, and canary feed mix. Captured rodents were transferred to the Parasitology Laboratory of the Life and Earth Science Department, Lebanese University - Faculty of Sciences in Fanar. Each rodent was identified to the species level, and its morphological traits, age, and sex were recorded.

#### a. Gastrointestinal sample preparation and observation

The rodents were put under anesthesia and then euthanized using ethyl ether. After this, their stomach, intestines, and cecum were removed and cut longitudinally. The contents of their gastrointestinal tract and any visible helminths were collected using a scalpel and dissection forceps. These were then stored in a 10 % formaldehyde solution to preserve adult parasites, larvae, and eggs. The samples were shaken using a vortex mixer to facilitate the detection of parasites and their eggs. Adult helminths were examined under a stereoscope, while small helminths, larvae, and eggs were studied under a light microscope at 40x magnification.

#### b. Fecal sample preparation and observation

The protozoan oocysts were collected using the flotation technique (Abi-Said *et al*, 2022). Briefly, fecal pellets were placed in a 3 ml solution of 30 % NaCl; then the tubes were agitated in a vortex mixer to homogenize the sample and release the oocysts. A few drops from each fecal sample were observed under a light microscope at 100x magnification.

### Identification of parasites

Endoparasites were identified based on their morphological traits at the Animal Research and Diagnostics Lab, Faculty of Agricultural and Food Sciences at the American University of Beirut (Tanideh *et al*., 2010; Koyama, 2013; Kim *et al*., 2015; Fitte *et al*, 2018; Maurelli *et al*., 2021).

### Statistical Analysis

Statistical analysis was estimated using the Statistical Package for the Social Sciences (SPSS) version 16.0 computer software program. A binary logistic regression model was used to test the possible relationship between sampling rodent species, gender, seasons, and site. Goodness-of-fit for each model was assessed using the Receiver Operating Characteristic (ROC).

## Ethical Approval

This research complied with all the relevant national regulations and institutional policies for the care and use of animals. We followed a protocol for handling animals conforming to the guidelines of the American Society of Mammalogists (Sikes *et al*. 2011). The protocol we followed includes measures designed to minimize stress and suffering for the animals involved in our study. We ensured that all procedures were conducted with the utmost care and respect for animal welfare, aligning with best practices in the field.

## Results

**Fig. 2.**
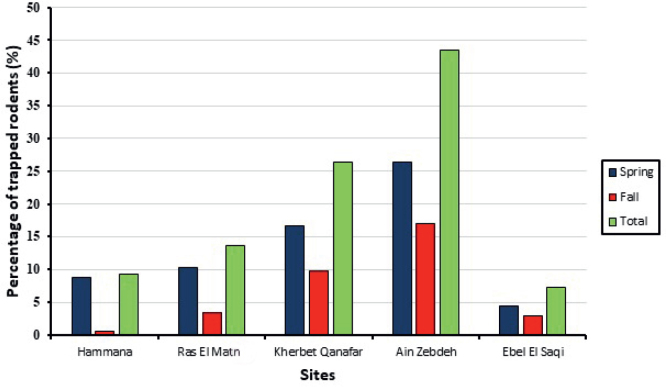
Percentage of trapped rodents in each studied area.

The 2,500 rodent trapping nights captured 205 rodents in Lebanon’s five Himas study areas. Most rodents (43.41 %) were trapped in Ain Zebdeh, while the fewest were trapped in Ebel El Saqi (7.32 %). Additionally, most rodents (66.34 %, N=136) were trapped during Spring (Fig. 2*)*.

**Fig. 3.**
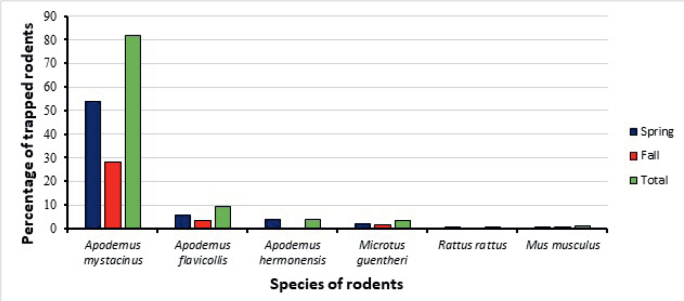
Percentage of trapped rodent species in different seasons.

The rodents captured in both seasons (Spring & Fall) included *Apodemus mystacinus, Apodemus flavicollis, Apodemus hermonensis, Microtus guentheri, Rattus rattus*, and *Mus musculus. A. mystacinus* was the most captured species (81.95 %), in both Spring (53.66 %) and Fall seasons (28.29 %). In comparison, *R. rattus* was the least captured species (0.49 %) (Fig. 3).

**Fig. 4.**
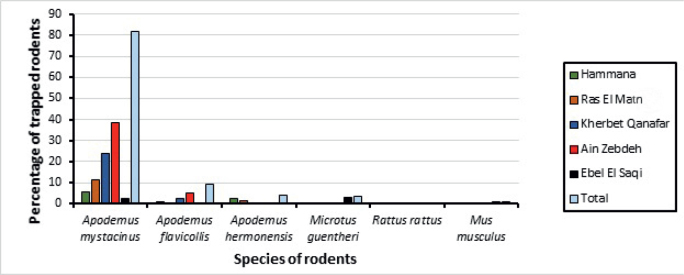
Percentage of trapped rodent species in different areas.

Furthermore, *A. mystacinus* and *A. flavicollis* were captured in all areas, with the highest percentage in Ain Zebdeh (38.54 %, N=79; 4.88 %, N=10, respectively). *A. hermonensis* and *M. guentheri* were captured in two areas, with the first being mostly captured in Hammana (2.44 %, N=5), and the latter in Ebel El Saqi (2.93 %, N=6). The rodents R. rattus and M. musculus were only trapped in Ebel El Saqi (N=2 and N=1, respectively) (Fig. 4).

## Intestinal parasites

Intestinal parasites isolated from trapped rodents included one cestode species, namely Hymenolepis diminuta, and eight nematode genera/species, including *Ascaris lumbricoides, Trichuris muris, Aspiculuris tetraptera, Heligmosomoides polygyrus, Syphacia muris, Syphacia obvelata, Capillaria* spp., and *Physaloptera* spp. Among the parasites isolated from the trapped rodents in the two seasons, nematodes were the most represented (82.4 %), while cestodes were the least represented (17.6 %). No protozoa were detected in the captured rodents in both seasons.

**Fig. 5.**
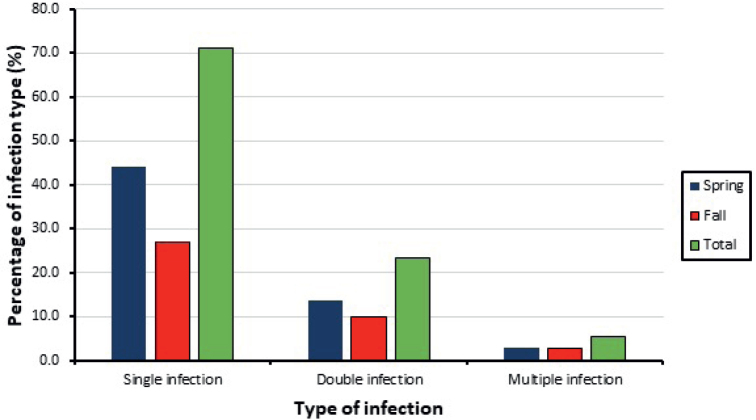
Percentage of infection type found in the trapped rodents.

Nearly half (54.1 %) of the captured rodents were infected with at least one intestinal parasite. Most infected individuals (71.2 %) were infected with a single parasite. Among the infected individuals, the percentage of those carrying one (44.1 %) or two (13.5 %) parasites was higher in the Spring. The infection with more than two parasites was similar in both seasons (2.7 %) (Fig. 5).

**Fig. 6.**
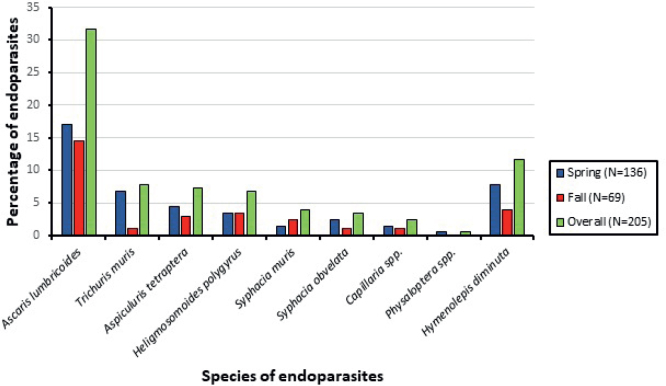
Percentage of collected endoparasites in the spring and fall seasons.

All the parasites were found in individuals trapped in both seasons, except for *Physaloptera* spp., which was only present in one individual trapped in Spring (Fig. 6). Moreover, most parasites were collected during the Spring season, including *A. lumbricoides* (17.1 %), *T. muris* (6.8 %), *A. tetraptera* (4.4 %), *S. obvelata* (1.5 %), and *H. diminuta* (7.8 %) (Fig. 6).

Species of rodents, gender, seasonality, and habitat had an influence on the intestinal parasites infecting rodents.

### a. Hymenolepis diminuta

*Hymenolepis diminuta* was the second most prominent parasite (11.7 %) among the trapped rodents (Fig. 6A). It was found in 14.3 % of the captured *Apodemus mystacinus* (Table 1). The total number of infected individuals with *H. diminuta* in the Spring (7.8 %) was more than those captured in the Fall (3.9 %) (Table 2). The comprehensive model assessing the factors influencing the infection of rodents with *Hymenolepis diminuta* accounted for 88.3 % of the variance, demonstrating a strong fit with a Receiver Operating Characteristic (ROC) value of 0.799. The location where the rodents were captured emerged as the most significant factor in determining the presence of *Hymenolepis diminuta* (Table 3).

**Table 1.** Percentage of captured rodents infected with specific helminth(s).

Parasite species	Host (rodent) species	Percentage of infected individuals
*Hymenolepis diminuta*	*Apodemus mystacinus*	14.3
*Ascaris lumbricoides*	*Apodemus mystacinus* *Apodemus flavicollis*	33.3 47.4
*Trichuris muris*	*Apodemus mystacinus* *Apodemus flavicollis*	7.7 21.0
*Aspiculuris tetraptera*	*Apodemus mystacinus* *Apodemus flavicollis* *Apodemus hermonensis* *Microtus guentheri*	5.9 15.8 12.5 14.3
*Heligmosomoides polygyrus*	*Apodemus mystacinus* *Apodemus flavicollis*	7.1 10.5
*Syphacia muris*	*Apodemus mystacinus*	4.2
*Syphacia obvelata*	*Apodemus mystacinus*	3.6
*Capillaria spp*.	*Apodemus mystacinus* *Apodemus flavicollis*	1.8 10.5
*Physaloptera* spp.	*Apodemus mystacinus*	0.6

**Table 2.** Percentage of rodents infected with parasites species in spring and Fall seasons.

Parasites		% of infected rodents	
	Spring (N=136)	Fall (N=69)	Overall (N=205)
*Ascaris lumbricoides*	17.1	14.6	31.7
*Trichuris muris*	6.8	1	7.8
*Aspiculuris tetraptera*	4.4	2.9	7.3
*Heligmosomoides polygyrus*	3.4	3.4	6.8
*Syphacia muris*	1.5	2.4	3.9
*Syphacia obvelata*	2.4	1	3.4
*Capillaria spp*.	1.5	1	2.4
*Physaloptera spp*.	0.5	0	0.5
*Hymenolepis diminuta*	7.8	3.9	11.7

**Table 3.** Impact of Season and Study site on rodent infection with specific internal parasites.

Parasites	Factor	B	S.E.	Wald	df	Sig.	Exp(B)
*Ascaris lumbricoides*	Spring	-0.665	0.340	3.821	1	0.051	0.514
	Site			21.834	4	0	
	Hammana	0.767	1.290	0.354	1	0.552	2.154
	Ras El Matn	0.183	1.275	0.021	1	0.886	1.201
	Kherbet Qanafar	1.820	1.082	2.829	1	0.093	6.174
	Ain Zebdeh	2.669	1.062	6.315	1	0.012	14.420
	Constant	-2.287	1.050	4.741	1	0.029	0.102
*Trichuris muris*	Spring	1.424	0.768	3.437	1	0.064	4.153
	Constant	-3.512	0.718	23.947	1	0.000	0.030
*Syphacia muris*	Spring	-1.655	0.850	3.789	1	0.052	0.191
	Constant	-2.549	0.464	30.143	1	0.000	0.078
*Hymenolepis diminuta*	Site			12.025	4	0.017	
	Hammana	19.881	1.038E4	0	1	0.998	4.308E8
	Ras El Matn	20.456	1.038E4	0	1	0.998	7.652E8
	Kherbet Qanafar	19.721	1.038E4	0	1	0.998	3.672E8
	Ain Zebdeh	16.726	1.038E4	0	1	0.999	1.836E7
	Constant	-21.203	1.038E4	0	1	0.998	0

### b. *Ascaris lumbricoides*

*Ascaris lumbricoides* was the most prominent parasite among the trapped rodents (31.7 %) (Fig. 6B). It was found in 33.3 % and 47.4 % of the captured *Apodemus mystacinus* and *A. flavicollis*, respectively (Table 1). The total number of infected individuals with *A. lumbricoides* in the Spring (17.1 %) was higher than those captured in the Fall season (14.6 %) (Table 2).

The comprehensive model assessing the factors influencing the infection of rodents with *Ascaris lumbricoides* accounted for 68.3 % of the variance, demonstrating a robust fit with an ROC value of 0.742. This indicates that the model effectively distinguishes infected and non-infected rodents. Among the various factors analyzed, the rodents’ capture location and the seasonal conditions were the most significant determinants of *Ascaris lumbricoides* presence (Table 3). Hence, rodents caught in the Spring were less likely to be infected by Ascaris lumbricoides, while rodents caught in Ain Zebdeh were most likely infected with *Ascaris lumbricoides*.

### c. *Trichuris muris*

**Fig. 7.**
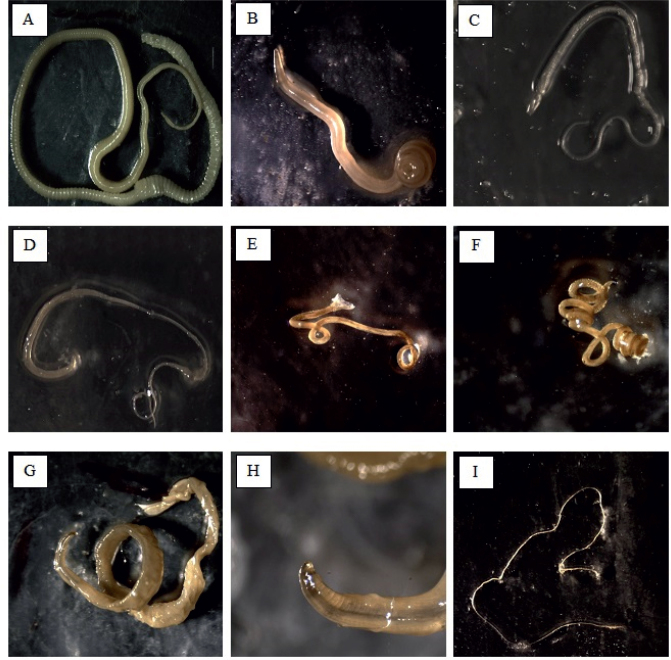
Intestinal helminths under stereoscope: (A) *Hymenolepis diminuta*, (B) Male *Ascaris lumbricoides*, (C) Female *Trichuris muris*, (D) Male *Trichuris muris*, (E) Male *Heligmosomoides polygyrus*, (F) Female *Heligmosomoides polygyrus*, (G) *Capillaria* spp, (H) *Physaloptera* spp., (I) *Physaloptera* spp. head.

*Trichuris muris* was found in 7.8 % of the trapped rodents (Fig. 7C, D). It was observed in two captured species: *Apodemus mystacinus* (7.7 %) and *A. flavicollis* (21.0 %) (Table 1). The total number of infected individuals with *T. muris* in the spring (6.8 %) was higher than that of those captured in the fall (1 %) (Table 2). The comprehensive model assessing the factors influencing the infection of rodents with *Trichuris muris* accounted for 91.7 % of the variance, demonstrating a robust fit with an ROC value of 0.619. The season tended to play the most important role in determining the presence of *Trichuris muris* (Table 3), whereby rodents caught in the Spring season tended to be more infected by this parasite.

### d. *Aspiculuris tetraptera*

**Fig. 8.**
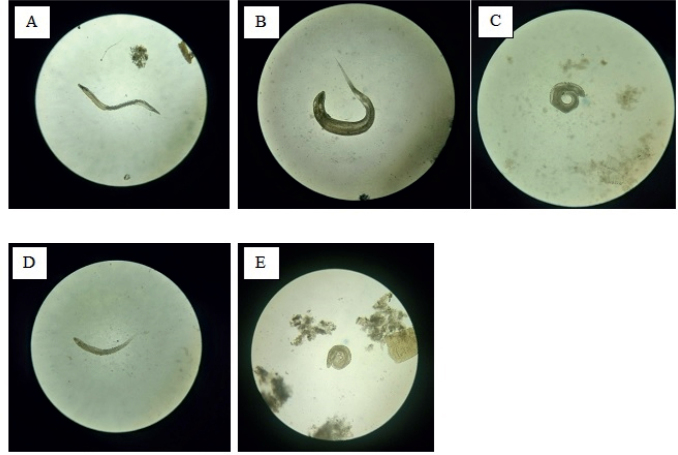
Intestinal helminths under the microscope (magnification 40 x): (A) *Aspiculuris tetraptera*, (B) Female *Syphacia muris*, (C) Male *Syphacia muris*, (D) Female *Syphacia obvelata*, (E) Male *Syphacia obvelata*.

The parasite *Aspiculuris tetraptera* was found in 7.3 % of trapped rodents (Fig. 8A). It was observed in four captured species of rodents: *Apodemus mystacinus* (5.9 %), *A. flavicollis* (15.8 %), *A. hermonensis* (12.5 %), and *Microtus guentheri* (14.3 %) (Table 1). The total number of infected individuals with *Aspiculuris tetraptera* in the Spring (4.4 %) was higher than those captured in the Fall season (2.9 %) (Table 2). Species of rodents, gender, season, and study site had no significant impact on the prevalence of *A. tetraptera* among the species caught (p>0.05).

### e. *Heligmosomoides polygyrus*

*Heligmosomoides polygyrus* was found in 6.8 % of trapped rodents (Fig. 7E, F). Around 7 % of the trapped *Apodemus mystacinus* and 10.5 % of *A. flavicollis* were infected with this specific worm (Table 1). The total number of infected individuals was equal in both seasons (3.4 %) (Table 2). Species of rodents, gender, season, and study site had no significant impact on the prevalence of *H. polygyrus* among the species caught (p>0.05).

### f. *Syphacia muris*

*Syphacia muris* was found in 4.2 % of the captured *Apodemus mystacinus* (3.9 % of the total trapped rodents (Fig. 8B, C, Table 1). The other captured rodent species did not harbor this worm. Rodents caught in the Spring season (1.5 %) were slightly less infected with *S. muris* than those captured in the Fall season (2.4 %) (Table 2). The overall model assessing the factors influencing the infection of rodents with *Syphacia muris* accounted for 96.6 % of the variance, and the model’s fit was deemed satisfactory with an ROC value of 0.696. The season tended to play the most important role in determining the presence of *Syphacia muris* (Table 3). Hence, rodents caught in the Fall season were more likely to be infected by *Syphacia muris*.

### g. *Syphacia obvelata*

This nematode was found in 3.4 % of trapped rodents (Fig. 8D, E). Only *A. mystacinus* individuals were infected with this helminth at a rate of 3.6 % (Table 1). The total number of infected individuals with *S. obvelata* in Spring (2.4 %) was slightly higher than those captured in Fall (1 %) *(*Table 2*)*. Species of rodents, gender, season, and study areas had no significant impact on the prevalence of *S. obvelata* areas among the species caught (p>0.05).

### h. *Capillaria* spp

*Capillaria* spp. was found in 2.4 % of the trapped rodents (Fig. 7G). It was observed in two rodent species, namely *Apodemus mystacinus* and *Apodemus flavicollis* (Table 1). The total number of infected individuals with *Capillaria* in the Spring season (1.5 %) was slightly more than those captured in the Fall season (1 %) (Table 2). Species of rodents, gender, season, and study site had no significant impact (p>0.05) on the prevalence of *Capillaria* spp. among the captured species.

### i. *Physaloptera* spp

*Physaloptera* spp. was found only in one rodent (*A. mystacinus*) in the Spring season in Ain Zebdeh (Tables 1, 2, Fig. 7H, I).

## Discussion

The number of rodents trapped during the Spring season was higher and more diverse than in the Fall. This is explained by the fact that this period coincides with their reproductive season (Yorulmaz & Albayrak, 2009). Moreover, the higher number of rodent species, mainly A. mystacinus and A. flavicollis, in Ain Zebdeh can be related to their proximity to the agricultural land in this site, ensuring a sufficient food supply, thus increasing the population size of these rodents. Similarly, Lema and Magige (2018) found that agricultural land was essential to the rodent community and had higher rodent diversity than forested areas in Tanzania. This was attributed to food availability and the heterogeneous environment in agricultural areas.

### a. *Hymenolepis diminuta*

*Hymenolepis diminuta* is a prevalent worldwide parasite with zoonotic potential, identified as the second most prominent parasite (11.7 %) among trapped rodents in this study. Although our work reported the presence of *H. diminuta* only in *Apodemus mystacinus*, various researchers have documented its presence in different rodent species in the Middle East, including *Apodemus fla*-*vicollis, A. sylvaticus, A. Agrarius, Mus musculus, Rattus rattus*, and *Rattus norvegicus* (Malla & Saida, 2022; Majede & Al-Amery, 2021a; Younis *et al*., 2021; Majede & Al-Amery, 2021b; Mohtasebi *et al*., 2020; Nabeel & Al-Tameemi, 2019; Abu-Madi *et al*., 2005; Gubanyi *et al*., 1992). Our findings of no gender-biased parasitism support previous studies (Malla & Saida, 2022; Archer *et al*., 2017) but contrast with others that found male-biased parasitism in urban settings of Lahore, Pakistan (Majeed & Al-Amery, 2021a; Ahmad *et al*., 2014), suggesting that ecological factors may influence these dynamics differently across regions. This variation was attributed to males moving from one community to another more frequently than females, thus having a greater chance of getting an infection from infected intermediate hosts.

The number of rodents infected with the cestode *H. diminuta* was not influenced by seasonal changes, indicating that its life cycle remains unaffected by climatic conditions at our study locations. In agreement with this finding, Ahmad *et al*. (2014) noted that infection rates of this cestode do not vary with seasonal fluctuations but are instead determined by the level of interaction between the intermediate host and the definitive host in rodent populations. The overall characteristics of the study sites did influence the presence of *H. diminuta*; this nematode was found in rodents across all trapping locations except for Ebel El Saqi, exhibiting varying prevalence (P<0.001) among these sites. The observed abundance of *H. diminuta* may indicate that the environmental conditions at our study sites are conducive to its survival and transmission, particularly due to the availability of its intermediate host, grain beetles, in these areas.

### b. *Ascaris lumbricoides*

The life cycle of the nematode species *Ascaris lumbricoides* is usually direct (Ransom & Foster, 1919) with zoonotic potential (Peng *et al*., 2007). *Ascaris lumbricoides* was the most (31.7 %) prominent parasite among the trapped rodents. Our findings also indicate that this helminth is reported for the first time as an infective agent in *Apodemus mystacinus* and *A. flavicollis*. Gender-biased parasitism was not observed in rodents infected with *A. lumbricoides*. This may reflect the equal susceptibility and exposure of both genders of the vector to this nematode (Smith *et al*., 2001). However, trapped rodents were most infected with *A. lumbricoides* in the Ain Zebdeh site during the Fall season. The seasonal and habitat differences in the infection rates can be attributed to the sensitivity of nematode transmission to climatic and habitat conditions. For instance, variations in temperature and humidity, as well as vegetation cover, affect the transmission of this parasite. During the fall season, leafy and root vegetables were available in Ain Zebdeh, an agricultural land, providing food resources that aided in the transmission of parasites. On the contrary, Fueki (1952) found that the infection of *Ascaris* from vegetables occurs more frequently in the spring when leafy vegetables are abundant in the market and the temperature is high enough for developing *Ascaris* eggs. The findings have highlighted that even the protected areas can facilitate the transmission of helminths due to the close interactions between wildlife and their ecosystems. For instance, rodents in these regions may harbor high prevalence rates of *Ascaris lumbricoides* due to factors such as abundant food sources and minimal disturbance from human activities. This prevalence is concerning because it suggests that these areas could act as reservoirs for the parasite, potentially leading to spillover infections in humans who come into contact with contaminated soil or water sources within these ecosystems.

### c. *Trichuris muris*

The nematode *Trichuris muris* has a direct life cycle, and it serves as a model of the gastrointestinal nematode *Trichuris trichiura* that infects humans (Marchiondo *et al*., 2019). Our findings agree with previous studies reporting the presence of this worm in *Apodemus* spp. It was reported across Europe in *Apodemus sylvaticus, Apodemus flavicollis, Mus domesticus*, and *Rattus rattus* (Callejón *et al*., 2010). In Kurdistan Province, West of Iran, *T. muris* was reported in the cecum of six types of rodents, including *Apodemus witherbyi, Microtus qazvinensis, Mus macedonicus, Dryomys nitedula, Meriones libycus*, and *Meriones persicus* (Mohammadi *et al*., 2022). The number of rodents infected with *T. muris* showed no variation among genders, which is consistent with the results of Kataranovski *et al*. (2011), who reported the absence of an effect of host gender on the parasitism for T. muris species in Rattus *norvegicus* in Serbia. Our results oppose those of Hepworth *et al*. (2010) and Hayes *et al*. (2007), who found that females are more resistant to infection. In contrast, males become chronically infected in Laboratory mice, which is attributed to the sex hormones, which are important factors in developing immunity in susceptible mice.

Seasonal variations significantly influence the prevalence of *T. muris* infections, with a notable increase in infected rodent populations during the Spring. This season creates conditions that are more convenient for the spread of *T. muris* eggs, as the life cycle of *Trichuris* spp. is adversely affected by harsh climatic factors such as intense sunlight, extreme temperatures, and insufficient moisture. Research indicates that optimal moisture levels and moderate temperatures are crucial for the survival and transmission of Trichuris eggs (Fahmy, 1954).

### d. *Aspiculuris tetraptera*

The nematode *Aspiculuris tetraptera* has a direct life cycle (Gerwin *et al*., 2017), and adult worms are typically located in the large intestine of mice (Whary *et al*., 2015). The prevalence of this parasite among four different rodent species shows no significant variation, suggesting that the infection is not specific to any particular species. This observation aligns with Behnke’s (1974) findings, which documented infections in the colons of various rodent species, including *Mus musculus, Rattus norvegicus*, and *Apodemus sylvaticus*. Additionally, there is no notable difference in infection rates between male and female rodents, supporting earlier studies (Kataranovski *et al*., 2008; Kia *et al*., 2010) that indicate equal susceptibility to this nematode across genders. However, our findings contrast with those of Li *et al*. (2023), who reported a male-biased parasitism in A. tetraptera infections among Brandt’s Voles (*Lasiopodomys brandtii*) in China, attributing this bias to males providing a larger ecological niche for parasitic invasion.

The season did not affect the number of rodents infected with this parasite. This could be explained by the absence of extreme variations between different seasons that affect the life cycle of *A. tetraptera* and its transmission (Li *et al*., 2021). In addition, the number of rodents infected with *A. tetraptera* did not vary among different trapping sites, probably due to the simple life cycle and the short incubation period of the *A. tetraptera* parasite. Likewise, Li *et al*. (2023) found no variation between habitat types (steppe and grazing) in infected Brandt’s Voles of China.

### e. *Heligmosomoides polygyrus*

*Heligmosomoides polygyrus* species has a direct cycle of infecting the small intestines of rodents (Marchiondo, 2019). The fact that our study reports the presence of this worm in rodents of the *Apodemus* spp. is in agreement with previous studies (Behnke, 2008; Eira *et al*., 2006). Furthermore, gender-biased parasitism was not observed in rodents infected with *H. polygyrus*, which agreed with the findings of Wertheim and Durette-Desset (1975). However, previous works reported a female-biased sex ratio in transmitting this parasite among rodents that may be caused by differences in immunity or host behavior (Ferrari *et al*., 2007; Wertheim and Durette-Desset, 1975). The fact that seasonality did not have any impact on the abundance of *H. polygyrus* in trapped rodents opposed the findings of Eira *et al*. (2006) that showed that the highest frequencies of *Apodemus sylvaticus* infection with *H. polygyrus* were reported during the humid Spring season. Moreover, Behnke’s (2008) observations reported a tendency of infection with H. polygyrus after periods of high humidity, where conditions become suitable for their development and transmission, which was not the case during our study.

### f. *Syphacia muris*

The nematode *Syphacia muris* has a direct life cycle (Lewis & D’Silva, 1986) and does not pose a zoonotic risk to humans (Lewis, 1968). Our research identified *S. muris* exclusively in the species *Apodemus mystacinus*. However, previous studies have documented the presence of this parasite in *Apodemus wetherbyi* in Iran (Mohammadi *et al*., 2022) and in black rats in Egypt (Menshawy *et al*., 2021).

Additionally, the prevalence of mice infected with *S. muris* was influenced by seasonal changes, showing a higher infection rate during the fall season. This finding is consistent with the observations made by Gomez Villafañe *et al*. (2008), who reported that *S. muris* was only found in *Rattus norvegicus* during the fall months in Argentina. The increased infection rates during this time are related to rat behaviors, particularly their grooming habits, which may facilitate the spread of the parasite.

### g. *Syphacia obvelata*

The life cycle of the nematode *Syphacia obvelata* is direct, primarily affecting the gastrointestinal tract of rodents and recognized as a potential zoonotic helminth (Whary *et al*., 2015; Riley, 1919). Our study observed that *S. obvelata* was present only in *Apodemus mystacinus*. Infection with this nematode has been documented in several rodent species, including brown rats, black house rats, house mice, lesser short-tailed gerbils, and both laboratory rats and mice (Khalil *et al*., 2014). Furthermore, our findings indicated no gender bias in parasitism among infected rodents. This aligns with the observations made by Yousefi *et al*. (2014), who also reported an absence of gender-biased parasitism in *Mus musculus* and *Apodemus sylvaticus*. The lack of disparity suggests that both male and female rodents have similar levels of exposure to and susceptibility to this nematode. Seasonality did not influence the number of rodents infected with *S. obvelata*. This shows that the life cycle of *S. obvelata* was not affected by the climatic conditions in our study sites. On the contrary, it was reported that the infection of male mice with *S. obvelata* in Gilloud Island was more pronounced during the warm season (the middle–end of the breeding season) (Pisanu *et al*., 203). The results were explained by endocrine-related increased host susceptibility, which was influenced by host age-related changes in the population rather than by host density-related factors.

### h. *Capillaria* spp

In this study, *Capillaria* spp. was observed only in rodents of the *Apodemus* spp. namely *A. mystacinus* and *A. flaviciollis*. In literature, *Capillaria* spp. was reported in the intestinal tract of various rodent species such as *Bandicota bengalensis, Bandicota indica*, and *Bandicota savilei* in Bangladesh (Fuehrer *et al*., 2012) and in the intestinal tract of *Rattus norvegicus* in Serbia (Kataranovski *et al*., 2011). Additionally, no evidence of gender-biased parasitism was found in rodents infected with *Capillaria* spp. This observation suggests that both male and female rodents captured in the study exhibit similar levels of susceptibility and exposure to this nematode. However, these findings contrast with those reported by Kataranovski *et al*. (2011), who identified a higher prevalence of *Capillaria* spp. infections in male *Rattus norvegicus*. They attributed this increased infection rate to the overlapping home ranges of males, which may lead to greater exposure to the parasite. At the same time, reproductive females tend to have a more defined site-specific organization that could result in lower transmission rates.

### i. *Physaloptera* spp

The genus *Physaloptera*, which belongs to the family Physalopteridae, comprises over 100 known species of parasites found worldwide. These parasites have an indirect life cycle, utilizing beetles, cockroaches, and crickets as intermediate hosts. Consequently, the distribution of these vector species influences the life cycle of *Physaloptera* spp. (Moradpour *et al*., 2018).

Our study observed that only one female *A. mystacinus* in Ain Zebde was infected with this parasite. This limited finding hinders our ability to evaluate how factors such as rodent species, gender, seasonal variations, and trapping locations affect the infection rate. Additionally, these results indicate a low prevalence of rodent infections with this specific parasite in Lebanese Himas.

### j. Protozoa

The absence of protozoan parasites such as Giardia, Toxoplasma, or Eimeria in our study’s rodent samples raises questions about the ecological and biological factors influencing parasite prevalence. Research indicates that different rodent species are susceptible to protozoan infections based on their habitat, diet, and immune responses. For instance, previous works have shown that particular species like *Rattus norvegicus* often harbor protozoa due to their scavenging behavior and close association with human environments (Chagas *et al*., 2017). In contrast, rodents inhabiting more isolated or less disturbed habitats may show lower infection rates. A study conducted in Qatar found that while some *Apodemus* species were hosts to various protozoa, others showed minimal or no infections (Islam *et al*., 2024). This suggests that environmental factors and host behavior significantly influence the presence of these parasites. A comprehensive study by Singla *et al*. (2008) noted that many wild rodents exhibit very low prevalence rates for protozoan parasites due to factors such as seasonal variations and habitat conditions. Furthermore, a study focusing on Lebanese wildlife rodents indicated that helminths are more commonly reported than protozoa among small mammals (Abi-Said *et al*., 2022). This aligns with our findings and suggests a trend where helminths dominate over protozoan infections.

It is worth noting that sample size and diagnostic approaches could influence the detection rates of protozoan parasites. Our study relied primarily on the floating technique without referring to molecular methods like Polymerase Chain Reaction; therefore, low-level infections with protozoa could have gone undetected.

## Conclusion

Nine intestinal parasite species were recovered from rodents captured in five different sites throughout Lebanon. These parasites included one cestode species, Hymenolepis diminuta, and eight nematode genera/species, including *Ascaris lumbricoides, Syphacia obvelata, Syphacia muris, Trichuris muris, Aspiculuris tetraptera, Heligmosomoides polygyrus, Capillaria* spp., and *Physaloptera* spp.

The four factors investigated for their effect on intestinal parasites in our study were the species of rodents, gender, seasonality, and study sites. Seasonality and sites played a significant role in influencing the abundance of intestinal parasites and infection. Temperature, humidity, and resource variation directly influenced the intestinal parasites’ survival and transmission, or indirectly by affecting the availability of their intermediate or definitive hosts. The absence of gender-biased parasitism and variation among rodent species reflects the susceptibility of both genders to the collected parasites and the absence of species-specific infection.

The influence of these factors on the dynamics of the parasites and their hosts reflects the importance of the stability of environmental conditions for the preservation of these dynamics and biological cycles. Therefore, any change in these conditions due to anthropogenic or natural activities might affect the populations of the parasites and the rodents. Anthropogenic activities and the growth in human populations are causing alterations to the environment in addition to the loss of rodent predators. Such actions are increasing rodent populations and thus increasing human-rodent contact, which consequently increases the chances of zoonotic disease, including intestinal parasites, to humans and other animals, thus threatening human health.

There are only a few studies on the variety of intestinal parasites observed in rodents in Lebanon. As a result, the findings of this study will further advance our understanding of rodent parasites and support future studies on endoparasites in Lebanon. More fundamental studies need to be conducted to determine whether any of the discovered species have a zoonotic potential that could harm human health in Lebanon.
